# The Education of the Public

**Published:** 1902-09-13

**Authors:** 


### THE EDUCATION OF THE PUBLIC.

Francis T. Bond, M.D. Lond., Hon. Secretary of the Jenner Society, writes:—

"I have no wish to criticise in any way your appreciation of the objects of the Imperial Vaccination League, but when you state that that body 'constitutes the first step ever taken in the country for the systematic instruction of the public upon a medical question,' I may be permitted, as a claim for bare justice, to point out that, apart from the work which the National Health Society and other associations which might be mentioned have been doing for many years past to educate the public on matters which are quite as medical in their character as vaccination is, the Jenner Society has for the last six years been engaged in carrying out precisely the same objects as those which the League is established to promote. During that time, with the encouragement and support of Lord Lister, of the Presidents of the Royal Society and of the British Medical Council, and with the valuable aid of the Council of the British Medical Association, the Jenner Society has directly or through the agency of Boards of Guardians, Sanitary Authorities and the medical profession, and the public generally, circulated many thousand copies of its publications, which have received the official sanction of the Local Government Board, and a list of which I enclose with this. If this has not been 'systematic instruction of the public' on the subject of vaccination I fail to see what else could be so designated, or how any other agency could otherwise accomplish this object."

